# A Brief Overview of Antenatal Care Services at Two Primary Care Centers in Bangladesh

**DOI:** 10.7759/cureus.104792

**Published:** 2026-03-06

**Authors:** Md Saifur Rahman, Mir Susmita Zaman, Mirza Md Asaduzzaman

**Affiliations:** 1 Obstetrics and Gynecology, Bhola 250 Bed District Sadar Hospital, Bhola, BGD; 2 Public Health, Pi Research and Development Center, Dhaka, BGD; 3 Gynecological Oncology, National Institute of Cancer Research and Hospital, Dhaka, BGD

**Keywords:** anc quality, antenatal care utilization, healthcare accessibility, health infrastructure, maternal healthcare

## Abstract

Background: The health and well-being of mothers and their fetuses depend on the timely utilization of antenatal care (ANC) and delivery services. Therefore, this study aimed to assess the current situation of ANC services at primary care centers in Bangladesh.

Methods: A cross-sectional study was conducted at two primary healthcare centers in Bangladesh over a six-month period. A total of 359 childbearing women who visited for ANC services were interviewed using a semistructured questionnaire. ANC service utilization was assessed in terms of the number of ANC visits, presence of health personnel, availability of iron tablets and folic acid, informing danger signs, and number of tetanus toxoid (TT) vaccinations, along with the overall satisfaction of participants during service utilization.

Results: The majority of participants were between 21 and 25 years old (37.6%), lived in semiurban (municipal area) areas (49.3%), and had completed at least secondary school (52.4%). Thirty-five percent of women were primigravid. Among participants, 55.7% had their first ANC visit. Only 45.1% women had completed the TT vaccination before the visit. Advice on family planning (72.4%) and eating nutritious food during pregnancy (82.5%) was received. Iron and folic acid tablets were accessible to 94.7% of the patients. During the ANC visit, privacy was maintained (96.4%), danger signs were addressed (83.6%), and the behaviors of healthcare providers were rated as very good (47.1%) or good (50.4%). Overall satisfaction with services was reported by 81.6% of participants.

Conclusion: The high level of participant satisfaction reflects the overall positive quality of primary health care services.

## Introduction

Antenatal care (ANC) refers to the care and services provided to women during pregnancy to ensure the well-being of both the mother and the child [1]. The quality of ANC plays a crucial role in improving maternal and neonatal outcomes [2]. Worldwide, 800 women die every day due to pregnancy and childbirth-related complications. About 287,000 maternal deaths occurred in 2020 worldwide, with nearly 95% reported in developing regions [3]. By 2030, the Sustainable Development Goals (SDGs) call for reducing maternal mortality worldwide to fewer than 70 deaths per 100,000 live births, sharing a commitment to safer motherhood [4].

Bangladesh has made considerable progress in maternal health, with maternal mortality declining by 38% between 2000 and 2022, from 441 per 100,000 to 156 deaths per 100,000 live births [5]. Evidence indicates that quality ANC can prevent many pregnancy-related complications and save lives [6,7]. It reduces risks of preterm birth and low birth weight and increases maternal use of skilled health professionals during delivery and postnatal care [8]. However, despite global improvements, service coverage remains insufficient. Also, respectful maternal care remained inadequate, as only 55% of women reported receiving respectful care during delivery. Notably, respectful maternal care was reported by 33.7% of women in public facilities compared with 72.8% in private settings [9]. In 2017, around 62% of pregnant women worldwide received at least four ANC visits as recommended by the World Health Organization (WHO), while in Bangladesh, the rate was only 47%, based on the 2017-2018 Bangladesh Demographic and Health Survey (BDHS) [10].

According to WHO, a minimum of four ANC visits is mandatory for a pregnant woman and her optimal care. Recently, eight or more ANC visits have also been recommended for a positive pregnancy experience [11]. Yet limited access to skilled providers and health facilities has left about 73% of Bangladeshi mothers without even four ANC visits, let alone the eight contacts suggested by WHO [12]. The composition of quality ANC may vary across settings; however, essential elements include nutritional interventions, clinical assessments, preventive and therapeutic measures, and system-level reforms to enhance the quality of care [11]. National surveys in Bangladesh have assessed ANC content through indicators such as blood pressure (BP) and weight measurement, laboratory testing, ultrasonography, and counseling regarding pregnancy complications and family planning. Among these, the first ANC visit is considered crucial because it identifies risk factors, estimates gestational age, and ensures early detection of potential complications [13]. However, a lack of timely initiation of ANC or no ANC can increase the risk for mothers and children [14]. Late ANC may lead to delayed diagnosis of complications, which might potentially detrimentally affect maternal and fetal health [15]. The availability and quality of technical ANC services, including ultrasonography and electrocardiography, are generally insufficient across many health facilities, especially in rural areas [16]. Although nearly 99% of health facilities in Bangladesh offer ANC services, only 4% are adequately prepared to deliver them in accordance with WHO standards [17]. Proper and timely ANC is linked with reduced maternal mortality, fewer pregnancy complications, and better overall health outcomes. Universal access to quality ANC services is therefore critical to ensuring healthier pregnancies.

Despite notable improvements, facility-level factors influencing ANC uptake remain insufficiently explored in Bangladesh, as in many other low- and middle-income countries. To address this gap, we conducted a study to assess the current status of ANC services in two selected primary health care centers in Bangladesh. The specific objectives of this study were to assess the availability and readiness of ANC services at the facility level, evaluate the utilization patterns of ANC services among pregnant women attending these facilities, and measure the level of patient satisfaction and identify factors associated with it. Evaluating the readiness and performance of these facilities in delivering ANC is essential for safeguarding maternal and fetal health. The insights generated from this study can inform maternal health planning, strengthen adherence to routine ANC, and support national efforts to achieve the SDG targets for maternal and neonatal well-being.

## Materials and methods

Study design, period, and locale

This facility-based cross-sectional study was conducted for six months, extending from January to June 2023, in two primary care centers of Bangladesh, Upazila Health Complex (UHC), Borhanuddin, and UHC, Daulatkhan. Both of these healthcare centers were located in the Bhola district of the Barishal division on the bank of the Meghna River. These facilities represent root-level government healthcare units serving rural and geographically vulnerable communities. These UHCs are the only place of healthcare available to these people. Existing data on ANC service quality, patients’ perceptions, available services, and satisfaction levels in these settings remain scarce. This study aimed to assess the availability of ANC services and measure the level of patient satisfaction and associated factors among pregnant women attending these facilities.

Study participants, sampling method, and sample size

The study population comprised pregnant women attending ANC services at the selected UHCs during the study period. Women were eligible if they 1) were currently pregnant, 2) were in any trimester (first, second, or third), 3) attended ANC services at either facility during data collection days, and 4) provided written informed consent. Women who were severely ill or unable to respond to the interview were excluded.

A purposive sampling technique was employed, whereby all pregnant women attending ANC services during the data collection period were approached and invited to participate until the desired sample size was reached. This approach was adopted due to the relatively low and variable patient flow in these rural facilities, and the study objective was to assess ANC services delivered at specific facilities, which required recruiting women who actually received care at those facilities.

The sample size was calculated using the single population proportion formula: $n = Z^2 \frac{pq}{d^2}$, assuming a 95% confidence level (Z = 1.96), 5% margin of error (d = 0.05), and an expected prevalence (p) of 47% for adequate ANC utilization based on the BDHS 2017-2018 [10,18]. After adjusting for a 10% nonresponse rate, the final required sample size was 382. A total of 382 eligible women were approached, of whom 359 completed the interview, yielding a response rate of 94.0%. Only completed questionnaires were included in the final analysis. Written informed consent was obtained from all participants prior to data collection.

Data collection procedure

The data were collected through face-to-face interviews using a paper-based semistructured questionnaire (see the Appendix). The interviews were conducted in a separate place after completion of the routine ANC services at the study sites. Participants were from semiurban areas, which are municipality areas of the upazila, or from rural areas outside the municipality of UHC, Borhanuddin, Bhola, and Daulatkhan upazilas.

The questionnaire was initially prepared in English and subsequently translated into Bangla to facilitate comprehension among participants. The translated version was reviewed by the research team to ensure clarity and contextual appropriateness.

A pretest was conducted among 20 pregnant women in a similar healthcare setting outside the study sites to assess clarity, comprehensibility, and flow of the questions. Based on feedback from the pretest, minor wording adjustments were made to improve understanding.

Two registered doctors working at the health care facilities collected the data for this study and received prior training on interview techniques, ethical considerations, and standardization of questionnaire administration. Regular supervision was conducted to ensure data quality and consistency by the principal investigator. Routine physical examination and laboratory investigations were performed according to standard guidelines. The detailed ANC measurements (BP, weight, hemoglobin, urine albumin, tetanus toxoid (TT) vaccination, and iron-folic acid supplementation) were collected with the questionnaire. Data on sociodemographic characteristics, obstetric history, service availability, and satisfaction levels were collected using predefined variables according to the WHO ANC recommendations, national guidelines, and previous studies.

Ethics statement

The ethical permission was obtained from the institutional review board of the Public Health Foundation (PHF-NG-1007). The privacy and confidentiality of information were ensured by limiting the data access to third parties and excluding participants’ names from the questionnaire. Written consent was obtained from study participants after being informed about the objectives, procedures, potential risks, and benefits of the study. Participation was entirely voluntary, and participants were informed of their right to withdraw at any stage without any consequences.

Statistical analysis

Data were entered into the IBM Statistical Package for the Social Sciences Statistics software for analysis (version 26.0; IBM Corp., Armonk, NY). Data cleaning was performed prior to analysis. Descriptive statistics were used to summarize the findings. Categorical variables were expressed as frequencies and percentages, while continuous variables were presented as mean ± standard deviation for normally distributed data. The factors associated with participant satisfaction were assessed using the chi-square test, and a p value of <0.05 was considered statistically significant.

## Results

Sociodemographic and personal information

This study included 359 pregnant women, of whom 37.6% were aged 21-25 years. Participants were mostly from semiurban areas (49.3%), while the rest (46.2%) were from rural areas. Half of the participants had completed higher secondary school (52.4%), and their monthly family income ranged from 10,000 to 20,000 taka. The respondents’ sociodemographic and personal information is depicted in Table 1.

**Table 1:** Characteristics of the study respondents (n = 359) The data are presented as the frequency and percentage, and within the sample size ^*^Age was also presented with mean ± SD SD: standard deviation; BDT: Bangladeshi Taka

Variables	Frequency	Percent
Age distribution		
Up to 20 years	90	25.1
21-25 years	135	37.6
26-30 years	105	29.2
31-35 years	22	6.1
Above 35 years	7	1.9
Mean ± SD (years)^*^	24.66 ± 5.62	-
Area of residence		
Semiurban	177	49.3
Rural	166	46.2
Maternal educational qualification		
Cannot read and write	7	1.9
Can read and write	10	2.8
Completed primary school	73	20.3
Completed secondary school	188	52.4
College and above	81	22.6
Monthly family income (in BDT)		
Less than 10,000	27	7.5
10,000 to <20,000	135	37.6
20,000 to <40,000	103	28.7
40,000 to <80,000	15	4.2
80,000 and above	1	0.3
Number of living children		
One child	138	38.4
Two or more children	79	22.0

Information related to ANC

About 34.5% women were primiparous among participants. About 26.3% had a history of abortion, and 12.0% had a history of stillbirth. Half of the women (55.7%) attended their first ANC visit. Iron and folic acid supplements for the current pregnancy were taken by 63.2% and 64.1% mothers, respectively. The majority (45.1%) of mothers had completed their TT vaccination. However, 3.9% had taken only one dose during pregnancy, while 20.9% remained unvaccinated (Table 2).

**Table 2:** Participant’s information related to ANC visit The data are presented as the frequency and percentage and within the sample size. Percentages are based on available responses for each variable due to missing data ANC: antenatal care; TT: tetanus toxoid

Variables	Category	Frequency	Percent
Gravida	First	124	34.5
	Second	115	32.0
	Third	89	24.8
	Four and more	31	8.6
History of abortion		94	26.3
History of stillbirth		43	12.0
Number of ANC in the current pregnancy	First	200	55.7
	Second	78	21.7
	Third	54	15.0
	Four and more	25	7.0
Supplemented with iron in the current pregnancy	Yes	227	63.2
	No	131	36.5
Supplemented with folic acid in current pregnancy	Yes	230	64.1
	No	127	35.4
TT vaccination status	Not vaccinated	75	20.9
	Vaccinated with one dose during pregnancy	14	3.9
	Vaccination completed	162	45.1
	Vaccinated, but could not tell the number of doses	107	29.8

ANC services at the primary care centers and their associated factors

The primary care centers were subjected to primary routine physical examination. Participants were asked about their last menstrual period, and the estimated delivery date was recorded (94%). Routine examinations, including the presence of any vaginal bleeding, fetal heart sounds, and routine laboratory investigations, were also performed. The UHC also advised the mother regarding family planning (72.4%), breastfeeding (73.5%), nutritious food (82.5%), personal hygiene (83.3%), vaccination of the child (75.5%), and follow-up visits (81.6%). Table 3 also shows the percentage of infrastructure available in primary care centers. Almost all the participants mentioned the facilities available at the UHCs. Participants who received ANC at these facilities were more likely to receive laboratory tests and physical exams than to receive counseling and health education. The most commonly received ANC services were diagnostic hemoglobin estimation (96%) and urine analysis (95%). A total of 94.2% of the participants reported the availability of TT injection facilities and the presence of all measuring tools during ANC visits (Table 3).

**Table 3:** Selected services/advice provided in antenatal care and available infrastructures of the centers The data are presented as the frequency and percentage within the sample size. Percentages are based on available responses for each variable BP: blood pressure; TT: tetanus toxoid; UHC: Upazila Health Complex

Variables	Category	Frequency	Percentage
Physical assessments	Measured height	350	97.5
	Measured weight	352	98.9
	Measured BP	353	98.3
	Checked Anemia	226	63.0
	Checked ankle edema	222	61.8
Availability of iron supplement		273	76.0
Assessed about last menstrual period		339	94.4
Informed about delivery date		329	94.0
Enquired about vaginal bleeding		213	59.3
Assessed fetal heart sound		224	62.4
UHC has advised the services regarding	Family planning	260	72.4
	Breast feeding	264	73.5
	Nutritious food during pregnancy	296	82.5
	Personal hygiene	299	83.3
	Vaccination of child	271	75.5
	Next visit	293	81.6
Infrastructure facilities available in the health care centers			
Sign board	Present	351	97.8
Time schedule board	Present	351	97.8
Waiting room	Present	325	93.1
Toilet for patients and attends	Present	348	96.9
Toilet condition	Satisfactory	332	92.5
Running water	Present	347	99.7
Facility for maintaining privacy	Yes	346	96.4
Presence of instruments	Sufficient	348	96.9
TT injection facility	Present	338	94.2
Height measuring scale	Available	348	96.9
Weight measuring machine	Available	348	96.9
BP machine	Available	349	97.2
Stethoscope	Available	349	97.2
Thermometer	Available	349	97.2
Ambulance	Available	348	96.9
Urine albumin detection facility	Available	341	95.0
Hemoglobin estimation facility	Available	348	96.9
Registration book records	Complete	335	93.3
Availability of iron and folic acid tablets	Available	340	94.7

**Figure 1:**
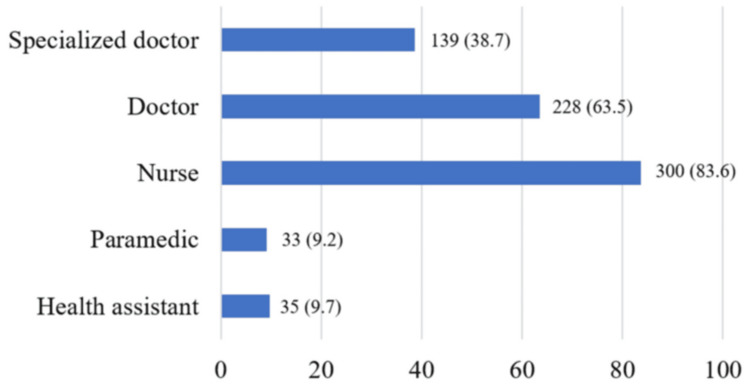
Available service providers of primary care centers

Figure 1 shows that nurses are key elements of reproductive health services, providing reproductive health services, which was higher than general practitioners and specialized doctors. Paramedics and health assistants also showed an inadequate number.

Participant satisfaction and service utilization

More than 90% of the participants were satisfied with the availability of service providers at the UHCs. The authors assessed the behaviors of healthcare providers as good (50.4%) or very good (47.1%), with 2.5% remaining neutral. Approximately 83.6% of the participants were informed about danger signs during pregnancy and what to do next. Approximately 81.6% of participants were satisfied, 15.3% were neutral, and 0.6% were not satisfied (Table 4).

**Table 4:** Information regarding services of the primary care centers The data are presented as the frequency and percentage within the sample size. Percentages are based on available responses for each variable due to missing data

Variable	Category	Frequency	Percentage
Availability of antenatal care service provider	Yes	345	96.1
	No	3	0.8
Behavior of health care provider	Very good	169	47.1
	Good	181	50.4
	Neutral	9	2.5
Information about danger signs during pregnancy	Given	300	83.6
	Not given	48	13.4
Overall satisfaction level	Satisfied	293	81.6
	Neutral	55	15.3
	Not satisfied	2	0.6

Assessment of factors associated with the satisfaction of the participant

Residency and receiver of TT vaccination during the pregnancy were found to be significantly associated with satisfaction levels, with p values of <0.001 and 0.002, respectively. However, other factors such as age group, gravida status, or number of ANC visits showed no significant association with the satisfaction level of the mother during their ANC services (Table 5).

**Table 5:** The associations of different factors with ANC service satisfaction of the participants ^*^p value was calculated with the chi-square test ^#^Values were presented as frequency and percentage within parenthesis ^a^Values were presented according to the availability of data ANC: antenatal care; TT: tetanus toxoid

Parameter	Overall satisfaction level^#^			Chi-square value	p value^*^
	Satisfied	Neutral	Not satisfied		
Age group					
≤25 years	188 (64.4%)	31 (56.4%)	1 (50%)	1.424	0.491
>25 years	104 (35.6%)	24 (43.6%)	1 (50%)		
Residency					
Semiurban	160 (55.6%)	9 (20.5%)^a^	1 (50%)	18.817	<0.001
Rural	128 (44.4%)	35 (79.5%)	1 (50%)		
Gravida					
Primi	103 (35.2%)	17 (30.9%)	0	1.351	0.492
Multi	190 (64.8%)	38 (69.1%)	2 (100%)		
Number of ANC visits					
Less than 4 visits	269 (92.1%)	54 (98.1%)	2 (100%)	3.326	0.246
4 or more visits	23 (7.9%)	1 (1.8%)	0		
Vaccination with TT in the current pregnancy	228 (77.8%)	50 (90.9%)^a^	0	12.625	0.002

## Discussion

This study aimed to understand the quality of ANC in Bangladesh and its determinants. The study highlights that the majority of pregnant women attending primary healthcare centers are within the optimal reproductive age and have achieved a reasonable level of education, with most residing in semiurban and rural areas. Our study revealed that the proportion of women with four or more timely ANC visits was considerably lower in comparison with higher proportions of participants with their first ANC visit. This suggests that many women may not have received clear or practical information regarding the nationally recommended schedule for ANC visits. Interestingly, evidence from Ethiopia shows an opposite pattern: while their first timely ANC visit rate is lower than what we observed, the proportion of women completing four timely ANC visits is substantially higher than in our setting [19]. This pattern suggests that the factors influencing timely initiation of ANC may not be the same as those that determine whether women continue and complete four timely ANC visits, and these determinants can vary across and within countries.

The age of a pregnant woman can significantly influence her willingness and ability to seek ANC services. In this study, both young (<20 years) and older (>35 years) mothers had a lower likelihood of accessing ANC services in Bangladesh. However, this is concerning because older pregnant women are more likely to face complications during pregnancy and childbirth and, therefore, should ideally have a higher uptake of such essential care [20]. In this study, women aged 21-25 years are more likely to undergo adequate ANC care, having more awareness and knowledge regarding ANC. According to Awasthi in Nepal, among 150 respondents, 51.4% were aged less than 25 years, and 39.4% were aged between 26 and 30 years seeking ANC care during their pregnancy [21].

Our study found that mothers who accessed ANC services were predominantly those with higher levels of education. This pattern has also been reported in studies from several other countries [22-24]. Evidence from Bangladesh similarly shows that education plays a crucial role in promoting adequate utilization of ANC [25]. Women with formal education tend to be more aware of their own health needs and the benefits of seeking care early in pregnancy. They are generally better informed about maternal health risks and are more motivated to use available services for their own well-being and that of their babies. Education also enhances a woman’s confidence and autonomy, enabling her to participate more actively in household decision-making, including choices related to healthcare. As a result, educated mothers are more likely to recognize the value of ANC and to follow recommended care schedules during pregnancy.

Among subjects, iron and folic acid supplementation, the key elements of ANC services, was sufficient. The TT vaccine was another crucial element. In this study, the majority of women (79%) in this trial had received the TT vaccination, compared with 3.9% who had received only one dose. Comparable results were observed among Nigerian women, of whom 72.9% had received at least two TT vaccinations [22]. Another study from Ethiopia showed similar results, with 59.6% of patients having completed two or more doses of the TT vaccine [23].

The results of the study demonstrate that both healthcare facilities have the basic infrastructure amenities, such as waiting areas, restrooms, billboards, and medical equipment, necessary to give treatment in a comfortable setting. A similar study is also present regarding the availability of basic infrastructure [26]. The majority of respondents stated that ANC service providers were available and had generally positive opinions of how healthcare professionals behaved. In this study, nurses play a pivotal role in delivering reproductive health services, although staff distribution is somewhat uneven, which may impact service delivery. However, both women and their families often prefer to seek care from physicians, particularly during the final trimester of pregnancy and at delivery [20]. Most respondents received information about danger signs during pregnancy, suggesting they were attempting to educate expectant mothers about potential risks. An efficient implementation of ANC services at the primary care centers is suggested by the high level of general satisfaction among respondents with the services given. Although the percentage of first prenatal care visits is excellent, efforts should be made to guarantee comprehensive ANC throughout pregnancy by encouraging and facilitating regular follow-up visits. Additional research has demonstrated that physicians serve as the main providers of ANC services [20,26].

Maternal mortality results from a complex interplay of factors, including social, economic, educational, political, and cultural influences, as well as issues such as gender inequality, inadequate infrastructure, challenging geographic conditions, and weaknesses in the health system. Evidence from Bangladesh and other regions shows that addressing only the health-related determinants, ensuring that all women have access to skilled and safe delivery services, can lead to a substantial reduction in maternal deaths [4]. However, this study provides valuable insights into various aspects of ANC utilization, service provision, and facility infrastructure among the target population by shedding light on the current status of ANC services and identifying areas for improvement in ANC services for primary care centers in Bangladesh. The findings can inform policymakers and healthcare providers about the existing gaps and strengths in ANC services, facilitating the development of targeted interventions and programs to enhance maternal healthcare delivery.

Despite these benefits, the study has several limitations. As a cross-sectional study, the data collected represent a snapshot of ANC utilization and service provision at a specific point in time in a specific place. This study design limited the ability to assess changes over time or to establish causal relationships. Another key limitation of this study is that it included only pregnant women who attended ANC services at the selected facilities, which may potentially introduce selection bias. However, the study provides valuable understandings regarding ANC utilization and service provision among the women who willingly attend the UHCs. Future research incorporating community-based sampling would be essential to capture the full spectrum of barriers to ANC utilization and provide a more comprehensive understanding of maternal healthcare access in Bangladesh.

## Conclusions

This study provides insights into the availability of ANC services and the experiences of pregnant women who utilized primary healthcare facilities in a rural setting of Bangladesh. The findings highlight gaps in continuity of care, particularly in completing recommended ANC visits, despite relatively good initial attendance and overall satisfaction among service users. These results suggest the need for targeted interventions within healthcare facilities to improve follow-up adherence, patient counseling, and service delivery. However, understanding the reasons why women delay or forget ANC requires mixed-methods research study. Such insights can guide policymakers, planners, and program designers to develop targeted interventions in the areas where they are most needed. Hence, continued exploration and monitoring are necessary to address the identified gaps and ensure the provision of high-quality ANC services to pregnant women and their newborns.

## Appendices

**Table 6:** Questionnaire BDT: Bangladeshi Taka; ANC: antenatal care; TT: tetanus toxoid; UHC: Upazila Health Complex; LMP: last menstrual period; BP: blood pressure

No		Questions	Responses	
Part I: Sociodemographic characteristics of respondents				
101		Age	________________________Years	
102		Residency	Semiurban Rural	
103		Religion	Muslim Hindu Christian Buddhist Other, specify________________________	
104		Marital status	Married Widowed Divorced Separated	
105		Mother's occupation?	Housewife Business Day labourer Employee Other, specify__________________	
106		Mother's educational status?	Can’t read and write Read and write Primary school Secondary school College and above	
107		Family monthly income	_______________BDT	
Part II: Questions related to factors associated with ANC utilization				
201	Gravida			First Second Third Four and more
202	Number of living children			0 1 2-4 ≥5
203	Did you have a history of abortion?			Yes No
204	Did you have a history of stillbirth?			Yes No
205	Number of ANC visits in the current pregnancy			First Second Third Fourth and more
206	Are you supplemented with iron during the current pregnancy?			Yes No
207	If the ans. for Q206 is Yes, for how long?			_____________days
208	Are you supplemented with Folic acid during the current pregnancy?			Yes No
209	Have you been vaccinated with TT during the current pregnancy?			Yes No
210	If the ans. for Q209 is Yes, how many times did you take TT?			___________
211	Did you miss or arrive late for your ANC visit?			Yes No
212	Reason (one or more) of missing or late to start ANC visit			Stay home principle Transport inaccessibility Unavailability of caregiver Negative health seeking behavior Peer pressure Any facility related reason Others If any specify_______________________
Part III: Questions related to the availability of instruments, investigations, medicines, and related facilities of the UHC				
Available healthcare facilities at UHC during ANC:				
301	Did anyone at the UHC measure height?			Yes No
302	Did anyone at the UHC measure weight?			Yes No
303	Did anyone at the UHC measure BP?			Yes No
304	Did anyone at the UHC see anemia in the eyelid?			Yes No
305	Did anyone at the UHC see edema in the ankle?			Yes No
306	Did anyone at the UHC supplied iron tablet?			Yes No
307	Did anyone at the UHC enquire about LMP?			Yes No
308	Did anyone at the UHC inform you about the expected delivery date?			Yes No
309	Did anyone at the UHC enquired per vaginal bleeding?			Yes No
310	Did anyone at the UHC measure uterine height?			Yes No
311	Did anyone at the UHC heard fetal heart sound?			Yes No
312	Did anyone at the UHC advice on a high-risk pregnancy?			Yes No
313	Did anyone at the UHC advice on family planning?			Yes No
314	Did anyone at the UHC advice on breastfeeding?			Yes No
315	Did anyone at the UHC advice on nutritious food?			Yes No
316	Did anyone at the UHC advice on personal hygiene?			Yes No
317	Did anyone at the UHC advice to vaccinate the child?			Yes No
318	Did anyone at the UHC advice on the next visit?			Yes No
Available facilities in the UHC:				
319	Sign board			Present Absent
320	Time schedule board			Present Absent
321	No. of ANC room			……………………………….
322	Waiting room			Present Absent
323	Toilets for patients and attendants			Present Absent
324	Toilet condition			Satisfactory Unsatisfactory
325	Running water			Present Absent
326	Facility for maintaining privacy			Yes No
Available instruments, investigations, medicines:				
327	Necessary instruments for ANC			Sufficient Insufficient
328	TT injection facility			Present Absent
329	Height measuring scale			Available Not available
330	Weight measuring scale			Available Not available
331	BP machine			Available Not available
332	Stethoscope			Available Not available
333	Thermometer			Available Not available
334	Ambulance			Available Not available
335	Urine albumin detection facility			Available Not available
336	Hemoglobin estimation facility			Available Not available
337	Record in the registration book			Complete Incomplete
338	Iron and folic acid tablets			Available Not available
Part IV: Availability of healthcare service providers				
401		Availability of the antenatal care service provider	Yes No	
402		Antenatal care service provider	Health Assistant Paramedic Nurse Doctor Specialized Doctor	
403		Behavior of the healthcare provider	Very good Good Bad	
404		Information given about danger signs during pregnancy	Yes No	
405		Overall satisfaction level of ANC services at UHC	Not satisfied Neutral Satisfied	
